# Asymptotic performance of the quadratic discriminant function to skewed training samples

**DOI:** 10.1186/s40064-016-3204-3

**Published:** 2016-09-13

**Authors:** Atinuke Adebanji, Michael Asamoah-Boaheng, Olivia Osei-Tutu

**Affiliations:** 1 Department of Mathematics, Kwame Nkrumah University of Science and Technology, PMB KNUST, Kumasi, Ghana; 2 Institute of Research, Innovation and Development (IRID), Kumasi Polytechnic, Box 854, Kumasi, Ghana; 3 Department of Mathematics, Kwame Nkrumah University of Science and Technology, PMB KNUST, 24105 Kumasi, Ghana

**Keywords:** Group centroid separator, Lognormal distribution, Error rates, Coefficient of Variation

## Abstract

This study investigates the asymptotic performance of the quadratic discriminant function (QDF) under skewed training samples. The main objective of this study is to evaluate the performance of the QDF under skewed distribution considering different sample size ratios, varying the group centroid separators and the number of variables. Three populations $(\pi _i, i=1, 2, 3)$ with increasing group centroid separator function were considered. A multivariate normal distributed data was simulated with MatLab R2009a. There was an increase in the average error rates of the sample size ratios 1:2:2 and 1:2:3 as the total sample size increased asymptotically in the skewed distribution when the centroid separator increased from 1 to 3. The QDF under the skewed distribution performed better for the sample size ratio 1:1:1 as compared to the other sampling ratios and under centroid separator $(\delta =5).$

## Background

Discriminant analysis is used in situations where the clusters are known a priori. The main aim of discriminant analysis is to classify an observation, or several observations, into these known groups (Härdle and Simar 2007). The problem of multiple group discrimination under normality and non-normality for a long time has posed a challenge to researches and several attempts have been made at deriving parsimonious rules that address this hurdle (Asamoah-Boaheng et al. 2014). This study evaluates the asymptotic performance of a three qroup quadratic discriminant function (QDF) under non-normal distribution with varying degrees of sample sizes, varying variable selections and under increasing group centroid separators.

Lachenbruch et al. (1977) studied the performance of the QDF under non-normality. They generated random samples from non-normal distributions and the samples were transformed into components by using Johnson’s system of transformation. Among their findings, they found that, the overall sample standard deviation, the between sample variability of the individual error rates of the function (QDF) under normal or non-normal distributions was quite large. In the computation of the overall sample standard deviation, the between sample variability of the individual error rates in the QDF on normal or non-normal distributions was quite large and for that instability of QDF is pronounced. Also the actual error rates were considerably larger than the optimal rates in the case of zero mean difference (this is a very difficult problem in assignment). The QDF for non-normal samples generally did not do substantially worse than when the QDF was derived under normal samples which were obtained after transformation. Lachenbruch et al. (1977) compared the re-substitution method and the leave-one-out method. The re-substitution method had an unacceptably high bias. The leave-one-out method was far superior in respect of generally having a far lesser bias.

Hosseini and Armacost (1992) presented a study on two group discriminant problem with equal group mean vectors with several methods and mathematical formulations. For comparative purposes, both Fishers linear discriminant function (FLDF) and that of QDF were used. Both methods performed better in the case of multivariate non-normal distributions than compared to that of the one generated from a multivariate normal distribution. All the various discriminatory methods performed better generally when the covariance matrices for the two populations were assumed to be unequal. Also, less favourable performance was observed for FLDF as well as QDF with presence of outliers than when there was absence of outliers/noise. Lachenbruch and Goldstein (1979) considered the effects of initial misclassification on the QDF. In his simulation, a population of two with equal priori probabilities, mean of 0 and 2 and number of variables, 2, 4, 8 and a fraction $\alpha _i$ of the $n_i$, which are actually from the other population, were considered. He then suggested that if initial misclassification is suspected, all sample points should be carefully checked and reassigned if needed. Krzanowski and Hand (1977) considered an assessment of error rate estimators paying special attention to the leave-one-out method. The estimator was investigated in a simulation study, both in absolute terms and in comparison with a popular bootstrap estimator. Motivated by this, extension of leave-one-out, the leave-two-out was looked at considering the variance. As expected, the leave-two-out method yields a slight variance reduction relative to the leave-one-out method, but was not enough to make it a good competitor.

In order to study the asymptotic error rates of linear, quadratic and logistic rules, Kakaï and Pelz (2010) conducted a Monte Carlo study in two, three and five-group discriminant analysis. The simulation study took into account the overlap of the populations ($e=0.05$, $e=0.1$, $e=1.5$), their common distribution (normal, chi-square with 4, 8 and 12 df) and their heteroscedasticity degree, $\Gamma$, measured by the value of the power function, $1-\beta$ of the homoscedasticity test related to $\Gamma$ ($1-\beta =0.05$, $1-\beta =0.4$, $1-\beta =0.6$, $1-\beta =0.8$). They found that the three rules gave similar error rates for normal homoscedastic populations. For non-normal populations, quadratic rule still gave lowest relative error except for two-group where logistic was the best. The quadratic and logistic rules were more influenced by the number of groups irrespective of their lowest relative error. Also linear and quadratic were more influenced by non-normality. The study deviates from Lachenbruch et al. (1977) by focusing on three populations, unequal sample sizes and log-normal distribution for the skewness. Croux (2004) studied the influence of observations on the misclassification probability in quadratic discriminant analysis. They also studied the effect of observations in the training sample on the performance of the associated classification rule. MacFarland (2001) investigated into the exact misclassification probabilities for plug-in normal quadratic functions; the case of equal mean. A stochastic representations for the exact distributions of the “plug-in” quadratic discriminant functions was derived for classifying a newly obtained observation.

As evident in the above literatures, several researchers have done extensive work on the performance of various discriminant and classification functions under skewed or non normal distributions. However, not much attention has been focused on studying and evaluating the performance of these classifiers using three populations under skewed distribution considering different sampling ratios, under different centroid separators and under varying variable selections. This study therefore seeks to investigate the performance of a single classifier (i.e the QDF) under skewed distribution considering different variable selections, varying sampling ratios and varying centroid separators considering three groups/populations.

## Methods

### The quadratic classifier ($\Sigma _1\ne \Sigma _2$)

Suppose that the joint densities of $X^{\prime }=[X_1,X_2,\ldots ,X_p]$ for population $\Pi _1$ and $\Pi _2$ are given by

1 $$ f_i(\mathbf {x})=\frac{1}{\left( 2\pi \right) ^{p/2}\left| \Sigma _{i} \right| ^{1/2}}exp\left[ -\frac{1}{2}({\mathbf {x}}-\mu _{i})^{\prime } \Sigma _{i}^{-1}({\mathbf {x}}-\mu _{i})\right] $$

When the multivariate normal densities have different covariance structures, the terms in the density ratio involving $|\Sigma _{i}^{1/2}|$ do not cancel as they do when we have equal covariance matrices and also the quadratic forms in the exponents of $f_i({\mathbf {x}})$ do not combine. Therefore substituting multivariate normal densities with different covariance matrices into Eq. (1) and after taking the natural logarithms and simplifying, the likelihood of the density ratios gives the quadratic function (assuming equal misclassification cost). Allocate **x** to $\Pi _1$ if

$$ -\frac{1}{2}{\mathbf {x}^{\prime}}\left( \Sigma ^{-1}_{1}-\Sigma ^{-1}_{2}\right) \mathbf {x}+\left( \mu ^{\prime}_{1}\Sigma ^{-1}_{1}-\mu ^{\prime}_{2}\Sigma ^{-1}_{2}\right) \mathbf {x}-k\ge \ln \left[ \left( \frac{p_2}{p_1}\right) \right] , $$

where

2 $$ k=\frac{1}{2}\ln \left( \frac{|\Sigma _1|}{|\Sigma _2|}\right) + \frac{1}{2} \left( \mu ^{\prime}_{1}\Sigma ^{-1}_{1}\mu _{1}-\mu ^{\prime}_{2}\Sigma ^{-1}_{2}\mu _{2}\right) $$

otherwise, ${\mathbf {x}}\in \Pi _2$. Considering the Mahalanobis distance, the function is sometimes written as

3 $$ f({\mathbf {x}})=D^{2}_{1}({\mathbf {x}})-D^{2}_{2}({\mathbf {x}})+ \ln \left[ \frac{|\Sigma _{1}|}{|\Sigma _{2}|}\right] -2\ln \left( \frac{p_1}{p_2}\right) $$

The quantity $D^{2}_{i}({\mathbf {x}})=(\mathbf {x}-\mu _{i})^{\prime}\Sigma ^{-1}({\mathbf {x}}-\mu _{i})$ is the Mahalanobis square distance.

When $\Sigma _{1}=\Sigma _{2}$ the function reduces to the linear classifier rule.

This function is easily extended to the three group classification where two cut off points are required for assigning observations to the three groups (Johnson and Wichern 2007).

### Simulation design

We evaluated the performance of QDF in case of skewed training samples following non normal distribution. In the simulation procedure, multivariate normally correlated random data was generated for three populations with their mean vector $\mu _1=(0, \ldots , 0)$, $\mu _2=(0, \ldots , \delta )$ and $\mu _3=(0, \ldots , 2\delta )$ respectively using MatLab R2009a.

The covariance matrices, $$\Sigma _i (i=1, 2, 3)$$, where $$k\ne l,$$$$\sigma _{kl}=0.7$$ for all groups except the diagonal entries given as $$\sigma _{k}^2 =i$$, for $$i=1,2,3$$ were obtained. Three different groups or populations which are normally correlated data were generated. Since the researchers were interested in evaluating the performance of the QDF under skewed uncorrelated data, the data was transformed from correlated normal to skewed data. In transforming the data, skewed data was generated by taking an exponents of the normally correlated/log normal data.

QDF was then performed in each case and the leave-one-out method was used to estimate the proportion of observations misclassified. Factors considered in this study were:

1. Mean vector separator which is set at $\delta$ from 1 to 5 where $\delta$ is determined by the difference between the mean vectors.
2. Sample sizes which are also specified. Here 14 values of $n_1$ set at 30, 60, 100, 150, 200, 250, 300, 400, 500, 600, 700, 800, 1000, 2000 and the sample size of $n_2$ and $n_3$ are determined by the sample ratios at 1:1:1, 1:2:2 and 1:2:3 and these ratios also determined the prior probabilities to be considered.
3. The number of variables were set at 4, 6 and 8 following (Murray 1977).
4. The size of population 1 $(n_1)$ was fixed throughout the study and the sizes of populations 2 and 3, $n_2$ and $n_3$ respectively are determined by the sample size ratio under consideration.

### Evaluating the performance of the QDF 

Let *r* denote the classification rule obtained on individuals belonging to *p*-variate populations with mixture density **F**. The error rate can be defined as the overall probability of misclassification associated with the classification rule. The probability $e_{jk}(r,{\mathbf{F}})$ that r allocates a random observation vector **X** to $G_{j}$ whiles it belongs to $G_{k}$ and is computed as follows [McLachlan (1992) as cited in Kakaï and Pelz (2010)].

$$ e_{jk}(r,{\mathbf{F}})= P( ({\mathbf{X}},{\mathbf{F}})=P( r({\mathbf{X}},{\mathbf{F}})=j|{\mathbf{X}}\in G_{k}),\quad (j, k=1,\ldots ,g-1;\quad j\ne k). $$

The overall error rate $e(r,{\mathbf{F}})$ associated with *r* is computed as shown below.

$$ e(r,{\mathbf{F}})= \sum \limits _{k=1}^{g}P_{k} \sum \limits _{j(\ne k)=1}^{g-1}e_{jk}(r,{\mathbf{F}}) $$

where $p_{k}(k = 1,\ldots ,g )$ is the group prior probability of $G_{k}$

## Results and discussion

This sections presents the outcome and discussion of the simulation results of the asymptotic performance of the QDF under skewed training samples.

### Performance of QDF under varying sampling ratios

**Fig. 1:**
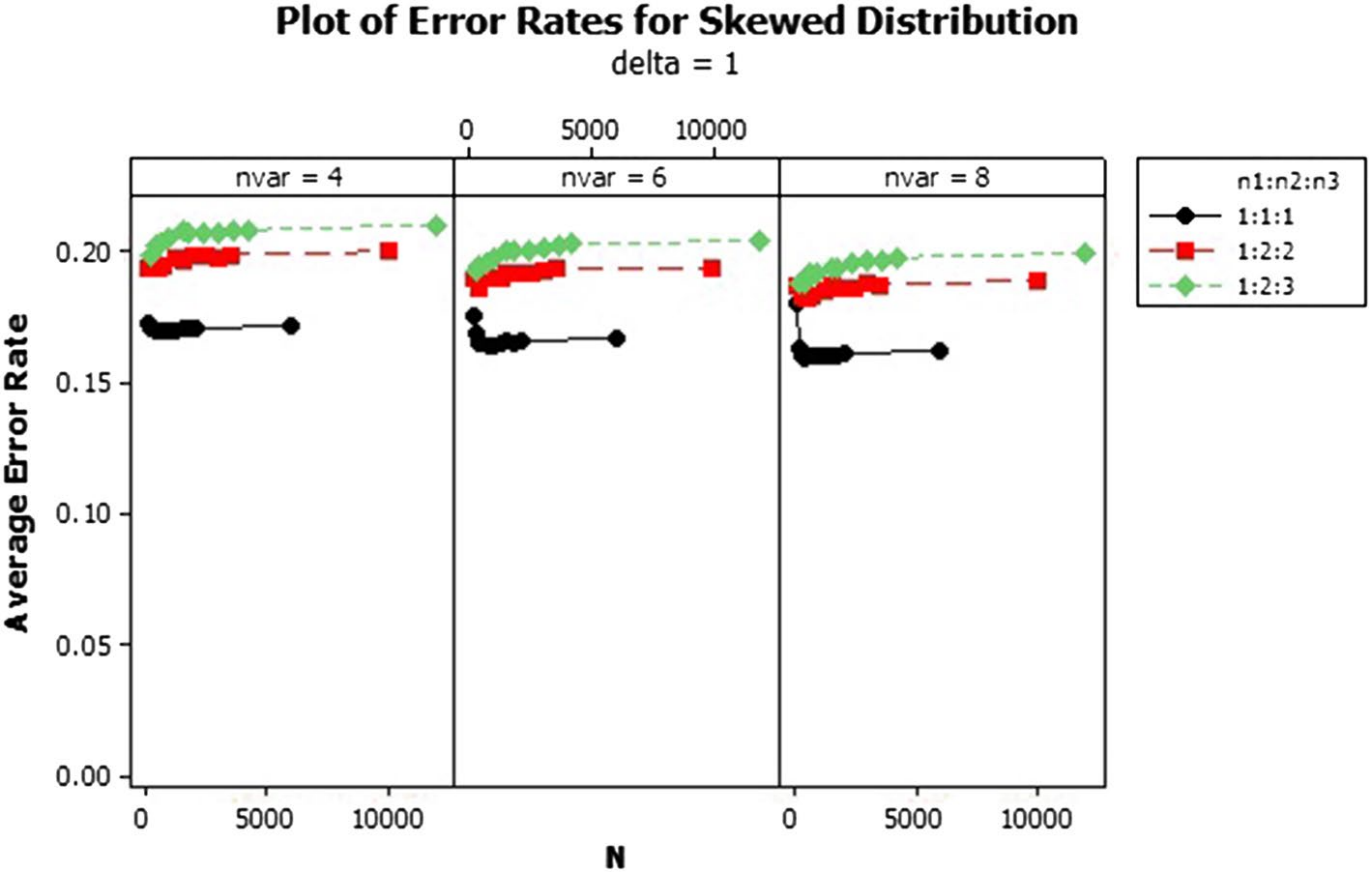
Average error rates of skewed distribution: $\delta =1$

**Fig. 2:**
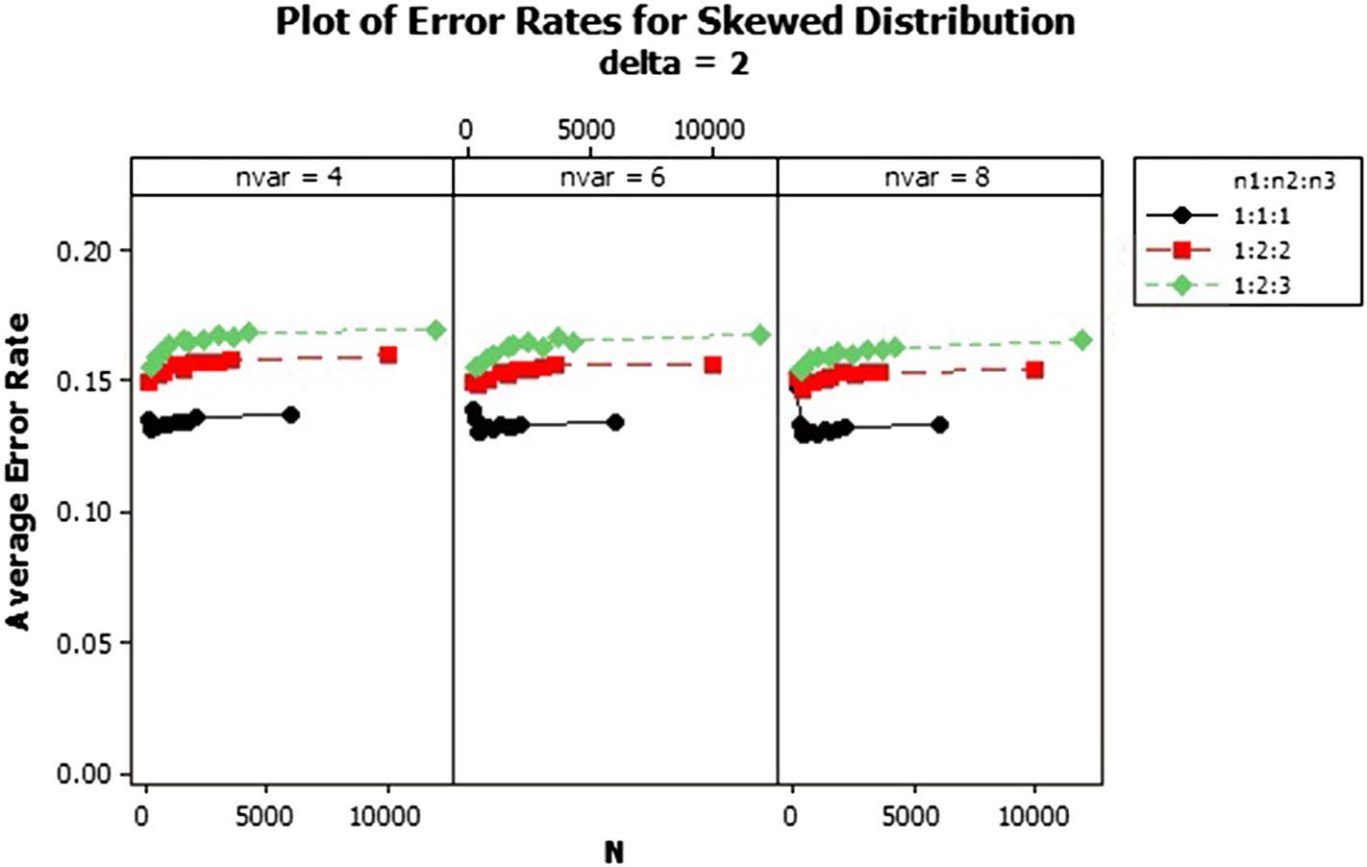
Average error rates of skewed distribution: $\delta =2$

**Fig. 3:**
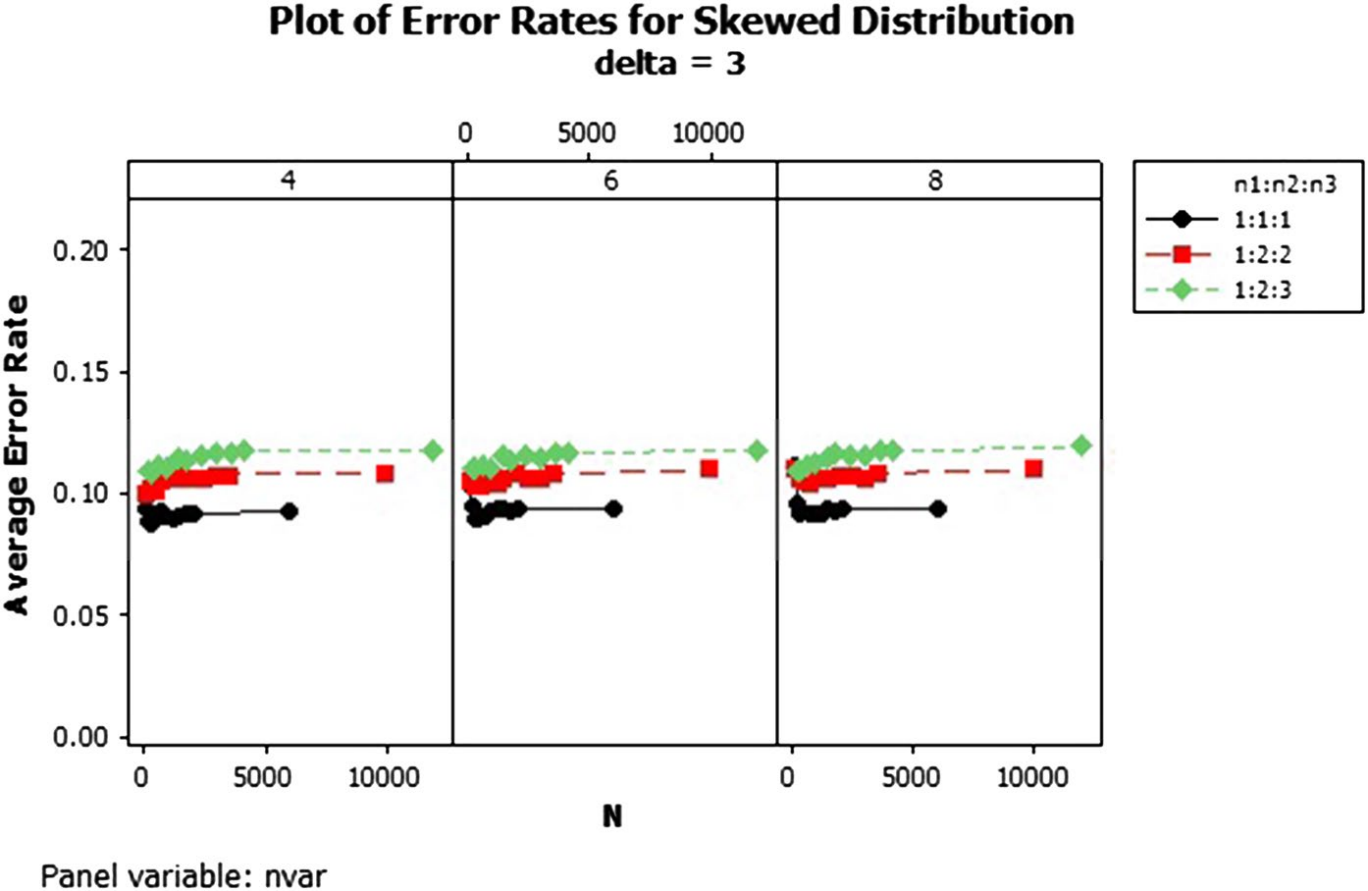
Average error rates of skewed distribution: $\delta =3$

From the results, there was an increase in the average error rates of the sample size ratios 1:2:2 and 1:2:3 as the total sample size increased asymptotically in the skewed distribution for $\delta$ = 1–3 as shown in Figs. 1, 2 and 3. In Fig. 1 for $\delta =1$ the lowest error rates were reported for equal sample size ratios (1:1:1). The error rates reduced marginally across the number of variables. Improvement in the performance was achieved with increased Mahalanobis distance and not asymptotically. The patterns of the error rates did not change significantly beyond $\delta =3$ as shown in Fig. 3. The average error rates for $\delta =5$ were the lowest as compared to the other $\delta$s and they decreased as the total sample size increased.

### Effects of number of variables on the performance of the QDF 

**Fig. 4:**
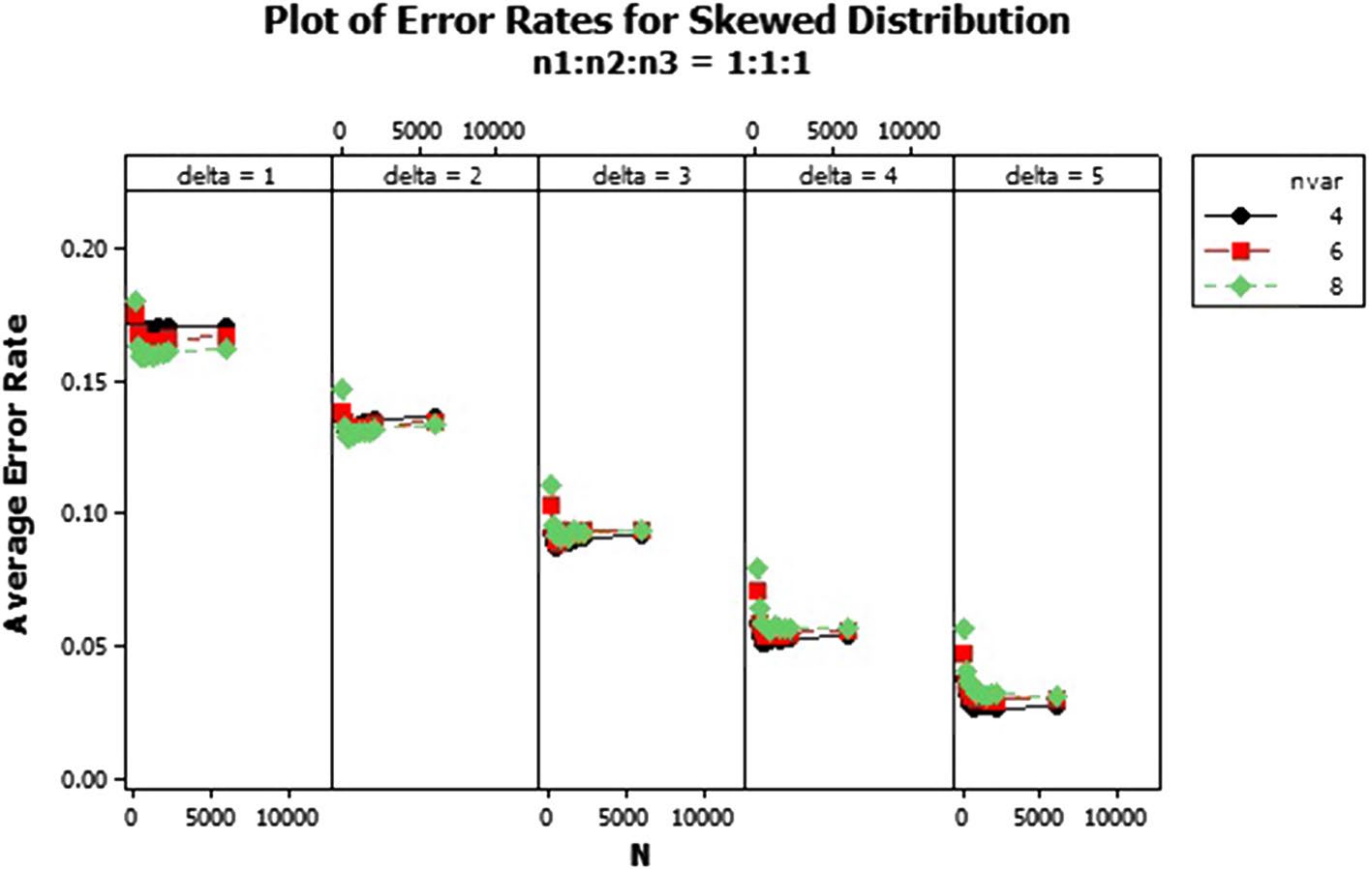
Average error rates of skewed distribution: $n_1:n_2:n_3=1:1:1$

**Fig. 5:**
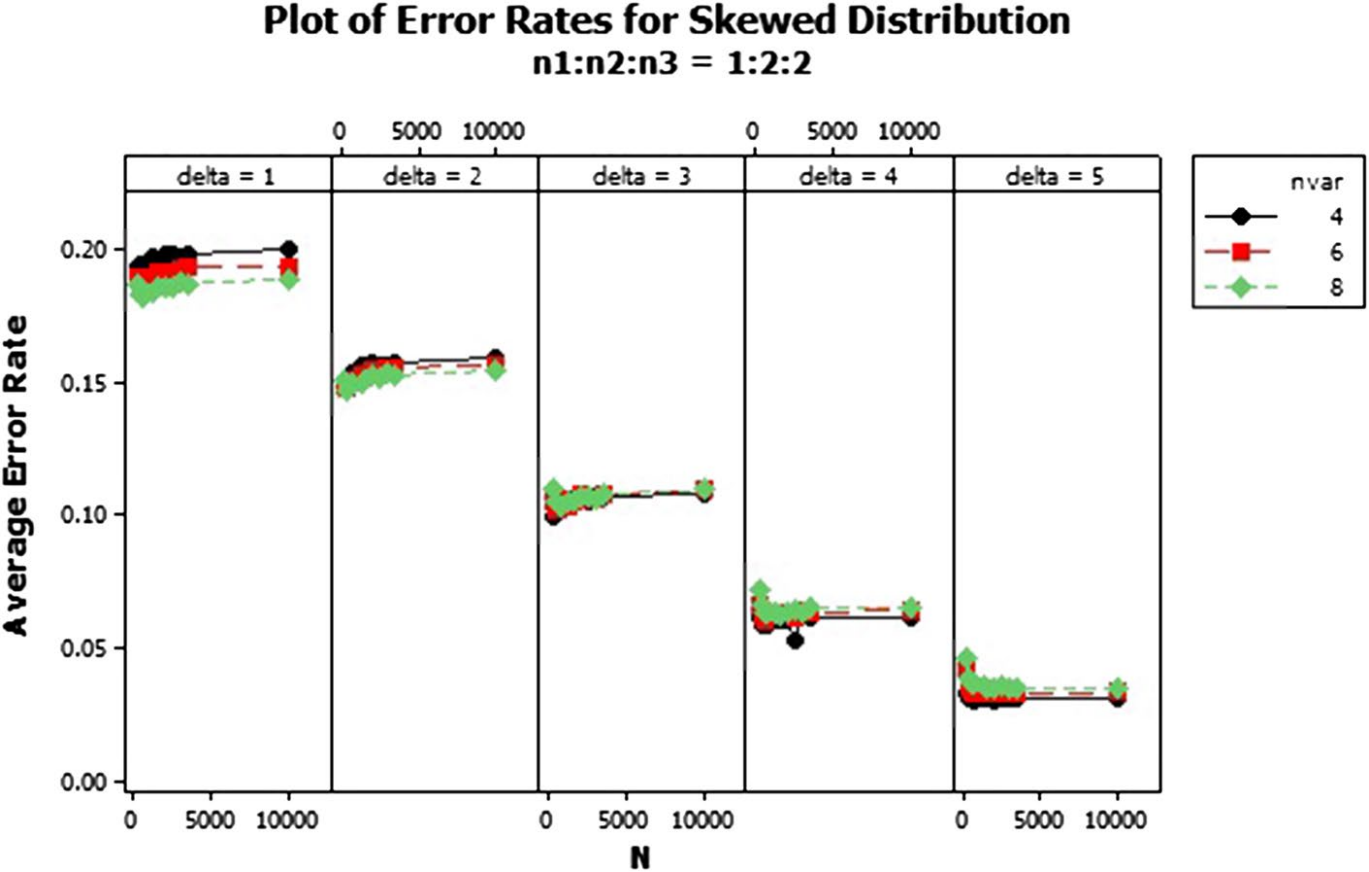
Average error rates of skewed distribution: $n_1:n_2:n_3=1:2:2$

**Fig. 6:**
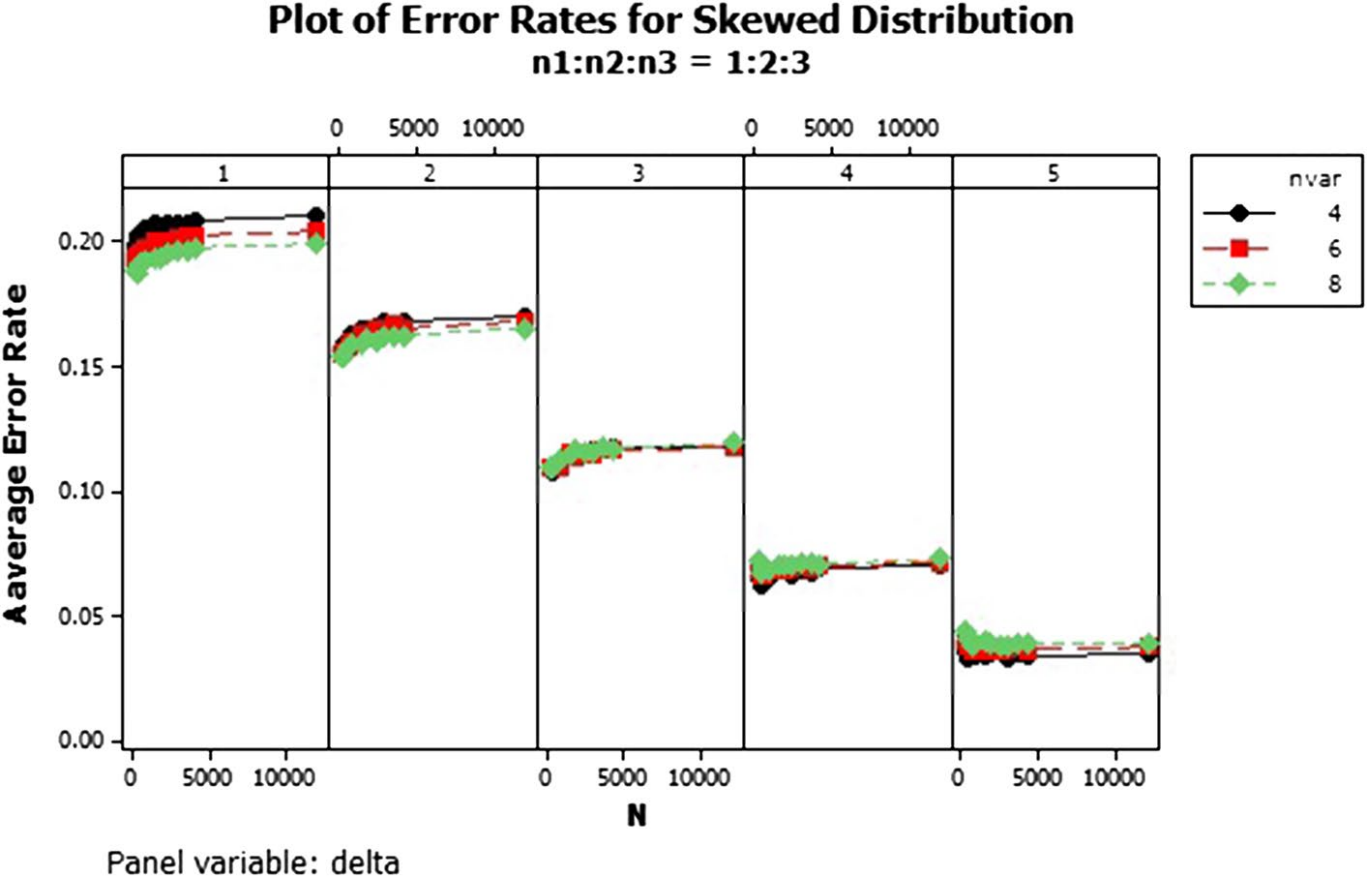
Average error rates of skewed distribution: $n_1:n_2:n_3=1:2:3$

The QDF performs differently with increasing number of variables. For sample size ratio 1:1:1, the average error rates of the variables reduced and curved upward as the total sample size increased for all $\delta$s, as shown in Fig. 4. The average error rates of sample ratios 1:2:2 and 1:2:3 were different as shown in Figs. 5 and 6. Also from Figs. 5 and 6 the average error rate of the QDF for the respective populations increased as the total sample size increased and reduced with increasing number of variable for $\delta =1$ and 2 . In $\delta =3$ and 4 of ratios 1:2:2 and 1:2:3, as the number of variables increased the average error rate of the QDF dropped from the total sample size of 150–300 and increased as the sample size also increased respectively while that of $\delta =5$ decreased marginally. In general the average error rate increased as the number of variables increased with increasing $\delta$.

### Effects of group centroid separator on the performance of QDF

**Fig. 7:**
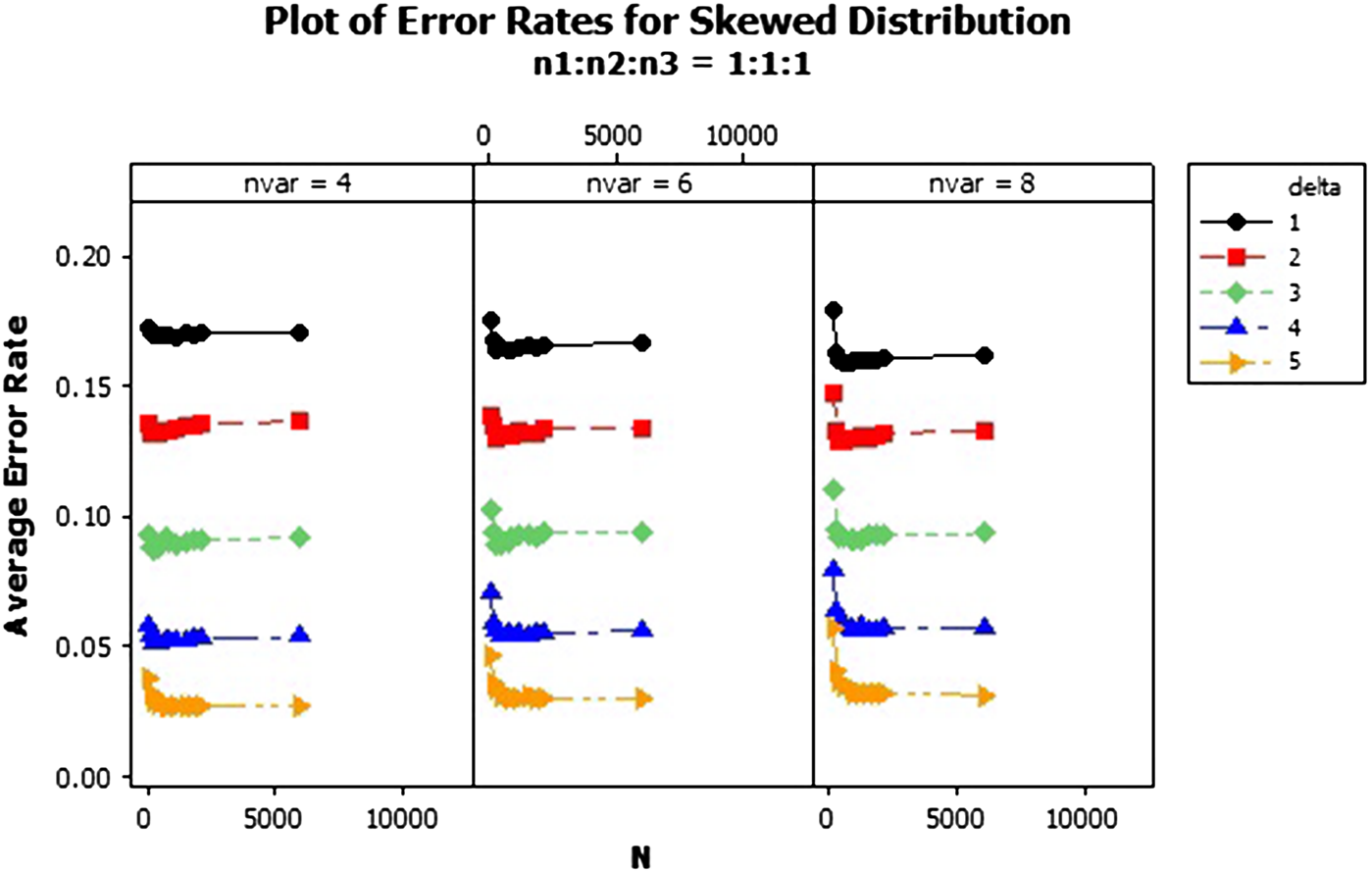
Average error rates of skewed distribution for $\delta$: $n_1{:}n_2{:}n_3=1{:}1{:}1$

**Fig. 8:**
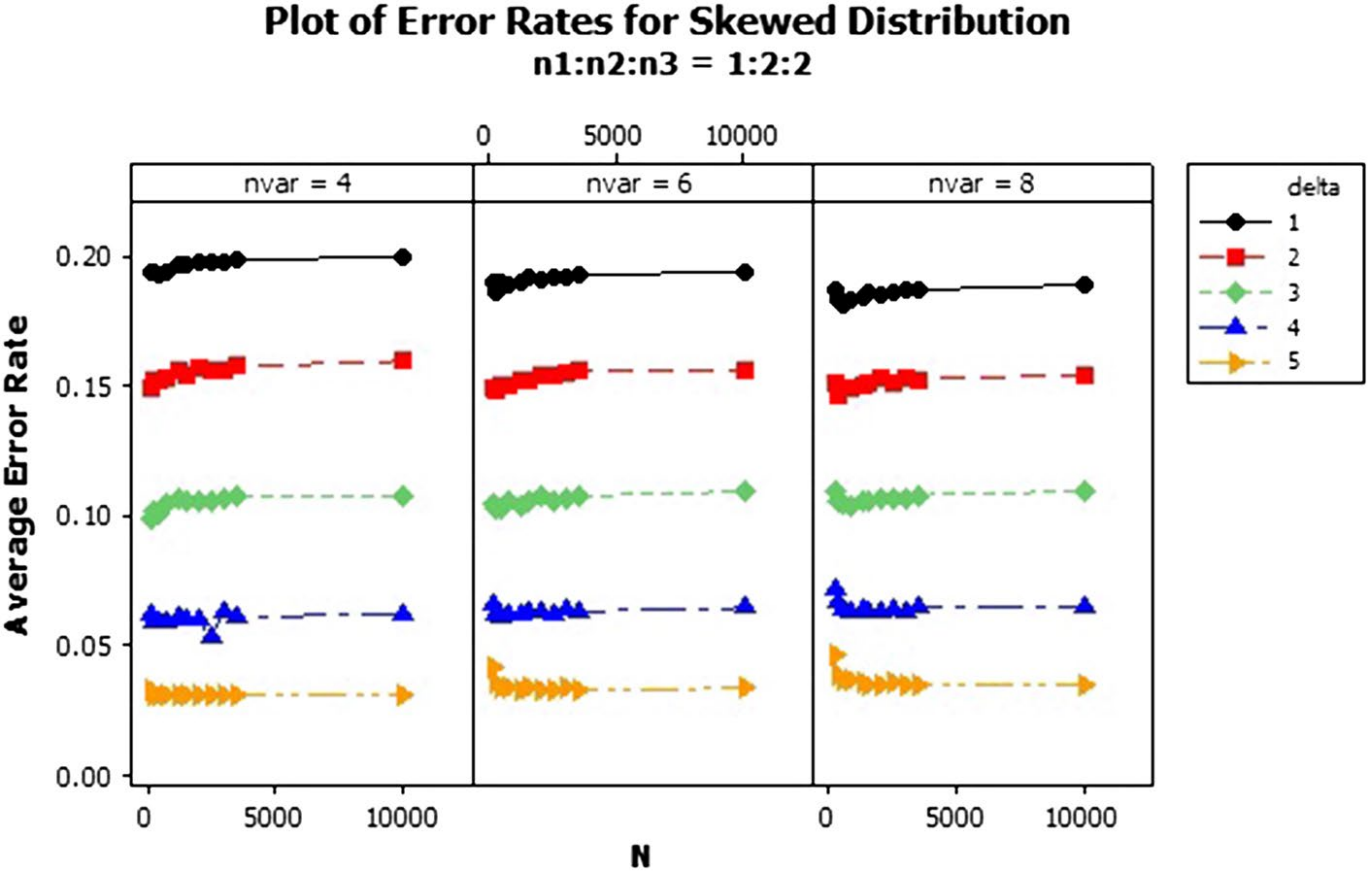
Average error rates of skewed distribution for $\delta$: $n_1{:}n_2{:}n_3=1{:}2{:}2$

**Fig. 9:**
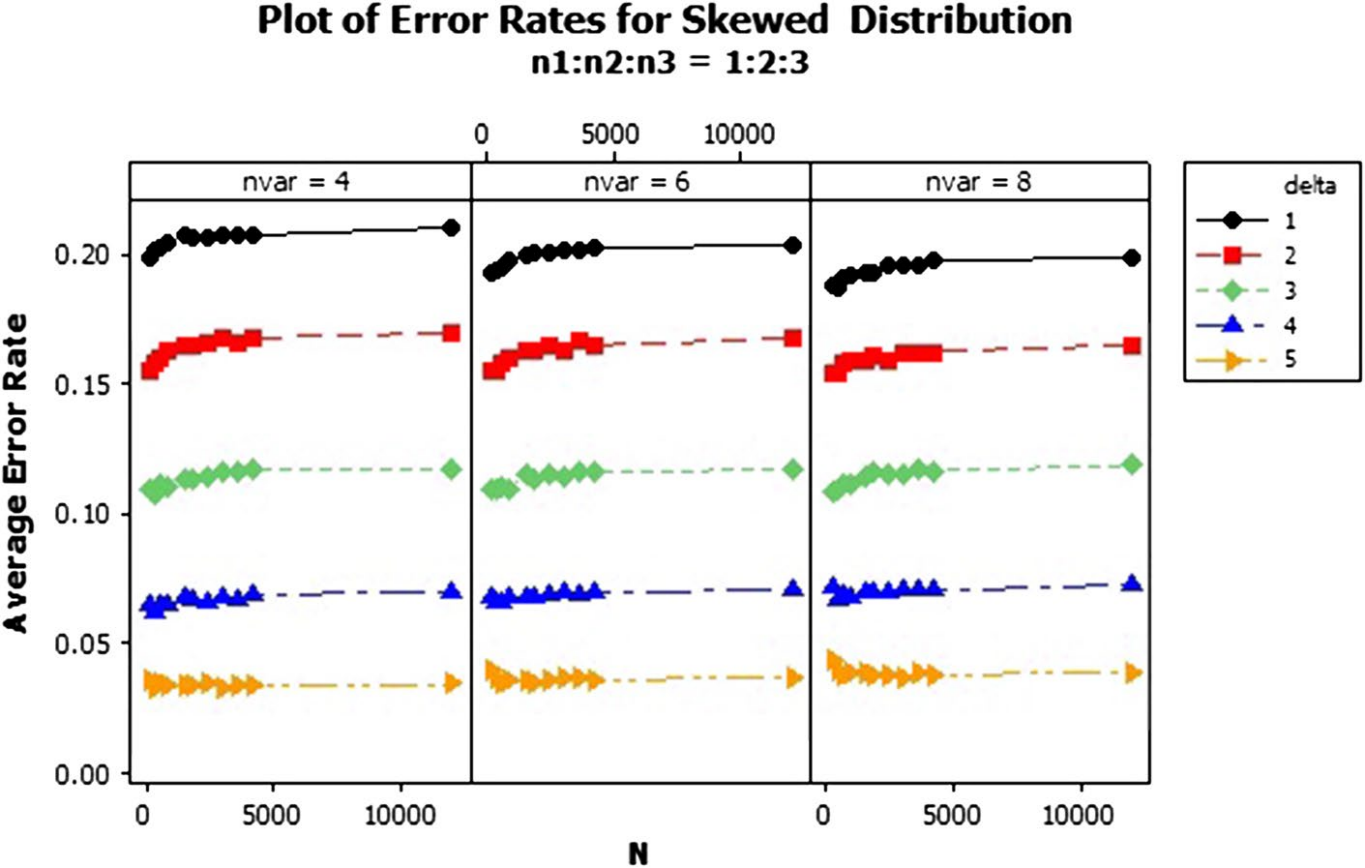
Average error rates of skewed distribution for $\delta$: $n_1{:}n_2{:}n_3=1{:}2{:}3$

The average error rate of the skewed distribution for sample size ratio 1:1:1 in Fig. 7 revealed that, as the sample size increases, the average error rates of the individual $\delta$s generally reduces. Also from Fig. 8, the error rates increased marginally for the individual deltas (centroids separators) as the sample sizes increases. However the performance of the QDF was quite abysmal when the centroid separator was set at $\delta =1$ as compared to the other deltas since it recorded the highest error rates with respect to each of the variable selections as 0.20. Also as clearly indicated in Fig. 8, the error rates of the QDF was minimised when the group centroid separator was set at $\delta =5$. Hence increasing the group centroid separators minimizes the misclassification rates thereby enhancing the performance of the QDF under the sample ratio of 1:2:2. Finally the performance of the QDF was evaluated under the sampling ratio of 1:2:3 with respect to the three groups/populations, $\pi _1, \pi _{2}, \pi _{3}$ with different selections of group centroids as shown in Fig. 9. From Fig. 9, similar results were obtained and the performance of the QDF was better under increasing group centroid separators, irrespective of the number of variables considered at a particular instance but was also dependent on the sample size selection.

## Conclusion

This paper investigated the asymptotic performance of QDF on skewed training data for three populations ($\pi _{i},i=1,2,3$) with increasing group centroid ($\delta$), with chosen variables and sample size ratios. Results from the study indicates that, the QDF performed quite poorly with an increase in error rates under sample ratios 1:2:2 and 1:2:3 for $\delta =1$–$\delta =3$. Other results also indicates that, the QDF performs better under an equal sample size ratio (1:1:1) resulting in a reduced misclassification rate with minimized error rates. The group centroid separators increased with decreasing group error rates and sample sizes. In other words, the QDF performed better in classifying the observations into their respective groups when the group centroid separators were increased. Also with increasing number of variables, from 4 to 8, the average error rate for evaluating the performance of the QDF dropped under $\delta =3, 4$ for sample ratios 1:2:2 and 1:2:3.

Generally, the study found that, there is always a pronouncement in the reduction of misclassification error rates as the group centroid separator increases as compared to an increasing sample size ratios. The results obtained from this study (skewed distribution) shows some conformity with Lachenbruch et al. (1977). Lachenbruch et al. (1977) generated random samples through simulations under non-normal distribution. Johnson’s system of transformation was used to transform the generated random samples into components by components. After the transformation, the QDF was derived and its performance was evaluated by the estimated mean error rates, standard deviation and sample variability. From their study the QDF recorded very high and increasing error rates, standard deviation under non-normality compared with the performance of the function under normally distributed data/training samples. In other words, they discovered that the QDF under non normal samples generally performs quite poorly as compared to when their performance are evaluated under normal distribution.
